# Short Social Media Videos as a Supplementary Educational Resource in Neuroanatomy: A Nonrandomized Clinical Trial

**DOI:** 10.1001/jamanetworkopen.2025.33971

**Published:** 2025-09-29

**Authors:** Bayan Alsaid, Ahmad Al-Bitar, Layla Mousa, Hossam Al-Mardini, Mhd Moanes Almaradni, Hisham Alhomsi, Dana Al-Masalma, Mohammad Bashar Izzat

**Affiliations:** 1 Laboratory of Anatomy, Faculty of Medicine, Damascus University, Damascus, Syrian Arab Republic; 2 Faculty of Medicine, Damascus University, Damascus, Syrian Arab Republic; 3 Department of Surgery, Faculty of Medicine, Damascus University, Damascus, Syrian Arab Republic

## Abstract

**Importance:**

The integration of microlearning tools, such as short social media videos, into medical education may offer novel strategies to enhance student engagement and knowledge acquisition. However, evidence regarding their effectiveness remains limited.

**Objective:**

To evaluate short-term academic performance and student engagement associated with short social media videos as a supplementary educational resource in a neuroanatomy course.

**Design, Setting, and Participants:**

This nonrandomized clinical trial was conducted at the Faculty of Medicine, Damascus University, during the 2024 to 2025 academic year. Third-year medical students who responded to an open invitation were enrolled and divided into an intervention group and a control group based on voluntary participation and access to the social media platform.

**Intervention:**

The intervention group engaged with 50 educational Instagram reels (each 90 seconds) over 2 months through a study-specific Instagram account. The control group continued traditional study methods without exposure to the videos.

**Main Outcomes and Measures:**

Primary outcomes included changes in neuroanatomy knowledge scores, measured by standardized preintervention and postintervention assessments. Secondary outcomes included student perceptions of the educational value and usability of the videos.

**Results:**

A total of 167 third-year medical students (84 men [50.3%]) were enrolled and divided into an intervention group (n = 84) and a control group (n = 83). Mean (SD) baseline assessment scores were 5.5 (2.8) of 20 in the intervention group and 5.5 (2.3) of 20 in the control group. Postintervention scores were significantly higher in the intervention group compared with the control group (mean [SD] score, 9.9 [2.1] of 20 vs 7.5 [2.4] of 20; *P* < .001). No significant differences were observed between the intervention group and the control group in final neuroanatomy examination scores (mean [SD] score, 17.2 [1.3] of 20 vs 16.5 [1.5] of 20; *P* = .09). Among intervention participants, 84.5% (71 of 84) reported that the videos were a beneficial supplement to traditional learning, although 27.4% (23 of 84) noted that the content delivery could feel monotonous.

**Conclusions and Relevance:**

In this nonrandomized clinical trial, the use of short social media videos was associated with improved short-term academic performance and positive student engagement but not with long-term benefit. The sustained impact of short social media videos demands ongoing evaluation, evidence-based modifications, and a focus on balancing creativity with educational outcomes.

## Key Points

**Question:**

Is the use of short social media videos as a supplementary educational tool associated with improved academic performance and student engagement in a neuroanatomy course?

**Findings:**

In this nonrandomized clinical trial of 167 medical students, short social media videos were associated with statistically significantly higher postintervention test scores compared with traditional learning methods alone. However, no significant difference was observed between Instagram reels and traditional learning methods in final neuroanatomy examination scores.

**Meaning:**

These findings suggest that Instagram reels may enhance short-term knowledge acquisition and engagement in medical education, but sustained academic benefits may require additional reinforcement strategies and careful content integration.

## Teaser

This nonrandomized clinical trial of 167 medical students in a neuroanatomy course evaluates the association of short social media videos as a supplementary educational tool with short-term academic performance and student engagement.

## Introduction

Anatomy education has evolved significantly from observation to dissection and currently incorporates digital media methodologies. Recently, online anatomical education content has proliferated on Facebook, X, Instagram, and YouTube.^1^ This advancement can supplement traditional laboratory and lecture-based anatomy teaching by providing students with convenient, affordable, and prompt access to digital anatomical images and virtual reconstructions. These innovative methods have also been helpful in addressing certain challenges, such as the need for retention of complex information,^2^ time constraints, and scarcity of experienced teaching staff.

Although anatomy educators are increasingly recognizing the value of integrating social media methodologies into formal curricula, this transition still requires an examination of the effectiveness of social media methodologies and their association with student engagement and learning outcomes.^3,4^ This study aimed to examine the usefulness of short social media videos as a supplementary educational resource and their association with academic achievements of students in the neuroanatomy course at the Faculty of Medicine, Damascus University.

## Methods

The study was registered and approved by the Biomedical Research Ethics Committees at Damascus University. Written informed consent was obtained from all participants before filling out the first questionnaire, with an emphasis on the confidentiality of information. All students were allowed to view the recorded clips after the end of the study period. This report followed the Transparent Reporting of Evaluations With Nonrandomized Designs (TREND) reporting guideline for nonrandomized trials.

### Study Design

This nonrandomized clinical trial was conducted during the academic year 2024-2025 among third-year medical students in the neuroanatomy course at Damascus University who volunteered to participate. A formal language proficiency test was not conducted, as all participants were advanced in their medical studies, where the curriculum uses Arabic for instruction and English for standard medical terminology. A high degree of bilingual proficiency was therefore assumed to be consistent across both groups.

Participants were recruited through an open invitation; students who registered their interest were further allocated based on their ability to access the intervention. A preparticipation questionnaire confirmed which students had an active Instagram account. Volunteers with an active account who consented to its use for the study formed the intervention group, which would watch the videos as a supplementary educational resource in neuroanatomy throughout 2 months, while a control group engaged solely in traditional learning methods without watching the videos. Adherence in the control group was based on self-reporting, as crossover viewing could not be systematically prevented due to the public nature of the social media platform.

To assess the baseline balance between the groups before the intervention, preparticipation data were analyzed. The groups were found to be highly comparable in terms of academic background. Previous anatomy course grades were similar (intervention group, 86.8 of 100; control group, 85.2 of 100), and the mean (SD) scores on the initial neuroanatomy knowledge assessment were 5.5 (2.8) of 20 in the intervention group and 5.5 (2.3) of 20 in the control group. Prior exposure to Instagram was an allocation variable rather than a confounder to be balanced, as it was a prerequisite for assignment to the intervention group.

Preparticipation data were collected for all participants, including gender, university grade point average, and previous anatomy course grades, as well as data on the use of social media. Additional information was collected from intervention groups related to the use of videos in the study, time spent on smartphones in general, and time spent on social media, especially Instagram.

### Content Creation and Intervention

A series of 50 educational short videos (90 seconds per video) covering the main topics in the curriculum of neuroanatomy was developed by the course instructor (B.A.), with technical assistance from the Damascus University technical team.

To ensure both factual accuracy and pedagogical value, the content creation and production process was conducted under the direct supervision of senior faculty from the Departments of Anatomy and Surgery (M.B.I). This internal review by subject matter experts served as the primary quality control measure prior to the reels’ release.

A dedicated Instagram channel was created for this study,^5^ and a new reel was uploaded daily over the 2-month study period (eFigure 1 in Supplement 1). Intervention group members were required to follow the account and interact with the videos as part of their study routine. Engagement was encouraged through regular reminders; however, direct, individualized monitoring of viewing metrics was not conducted to protect student privacy.

Individual engagement (eg, views, likes, watch time) was not objectively tracked through platform analytics. The study relied on self-reported engagement instead. Students in the control group were asked to engage solely in traditional learning methods without watching the videos during the study period.

### Students’ Performance Evaluation

Each participant was required to take a test via Google Forms twice, immediately before the study started and 2 months later, just before students began preparing for formal university examinations. A dedicated questionnaire was developed to evaluate students’ knowledge of the main topics in the curriculum of neuroanatomy. Twenty questions of various types (single-answer multiple-choice questions, multiple-answer multiple-choice questions, and the use of drawings with arrows pointing to anatomical elements) were used. Ten questions were in Arabic and 10 were in English (eFigure 2 in Supplement 1). Finally, participants were asked to express their views regarding the effectiveness of the videos as an educational tool, including certain aspects such as interaction, understanding, and information retention.

### Statistical Analysis

Paired *t* tests were used to compare preparticipation and postparticipation knowledge scores within the intervention and control groups to assess changes in academic performance. These tests were 2-sided, with a significance level set at *P* < .05. Prior to performing these analyses, the normality of data distribution for all score variables was assessed through visual inspection of histograms and Q-Q plots and confirmed using Shapiro-Wilk tests, indicating sufficient normality for the use of parametric tests. To compare differences between the intervention and control groups at each time point (preparticipation, postparticipation, and final university examination scores), Mann-Whitney *U* tests were used.

Survey responses regarding student perceptions and satisfaction were analyzed using descriptive statistics (frequencies and percentages). Inferential tests, such as χ^2^ tests, were used to determine trends and associations in survey data. In addition, a multiple linear regression analysis was conducted to investigate the factors associated with student performance (current neuroanatomy scores) beyond the direct intervention effect. The dependent variable was the student’s current score in neuroanatomy. Independent variables included the study group (intervention vs control), gender, previous anatomy course grades, and university grade point average. Assumptions of the regression model, including linearity, independence of residuals, and homoscedasticity, were assessed prior to analysis. All statistical analyses were performed using IBM SPSS Statistics software, version 26 (IBM Corporation). No specialized extension packages were used for this analysis.

## Results

Two hundred students registered their interest in joining the study, and 167 students (84 men [50.3%] and 83 women [49.7%]) completed the study, constituting the study group. The intervention group consisted of 84 students and the control group consisted of 83 students. Preparticipation demographics and questionnaire results are presented in Table 1 and Table 2. More than half the participating students (100 [59.9%]) already had active social media accounts, even though most videos were being watched for entertainment purposes.

**Table 1.** Preparticipation Demographic Characteristics

Characteristic	Students, No. (%)		
	Total study (N = 167)	Intervention (n = 84)	Control (n = 83)
Gender			
Female	83 (49.7)	42 (50.0)	41 (49.4)
Male	84 (50.3)	42 (50.0)	42 (50.6)
Use of social media^a^			
Active accounts	100 (59.9)	73 (86.9)	27 (32.5)
Inactive accounts	61 (36.7)	11 (13.1)	50 (60.2)
No account	6 (3.3)	0	6 (7.2)
Use of Instagram			
Active accounts	92 (55.1)	73 (86.9)	19 (22.9)
Inactive accounts	45 (26.9)	11 (13.1)	34 (41.0)
No account	30 (18.0)	0	30 (36.1)
Use of Facebook			
Active accounts	73 (43.7)	58 (69.0)	15 (18.1)
Inactive accounts	72 (43.1)	26 (31.0)	46 (55.4)
No account	22 (13.2)	0	22 (26.5)
Total grade point average and previous anatomy course grades			
Total grade point average	83.5/100	83.6/100	83.3/100
Anatomy course	86.0/100	86.8/100	85.2/100

^a^ Social media use: active account, student owns and uses the platform regularly (ie, multiple times per week); inactive account, student has an account but uses it rarely or not at all; and no account, student does not own or has never registered an account on the platform.

**Table 2.** Preparticipation Questionnaire Results From the Intervention Group

Questionnaire item	Students, No. (%) (N = 84)
Time spent on phone screen, mean (SD), min/d	
On mobile phones	586 (575)
On social media	117 (81)
Watching videos	
For entertainment	62 (73.8)
For education	22 (26.2)
Can video reels help in scientific topics?	
Yes	80 (95.2)
No	4 (4.8)
Do you prefer reels created by teaching staff?	
Yes	63 (75.0)
No	21 (25.0)

### Students’ Performance Outcomes

The mean and median scores for both groups were calculated at 3 time points: preintervention, postintervention, and the final university examination. The intervention group showed a more substantial increase in scores after the 2-month study period. For the intervention group, the pretest scores showed a mean (SD) of 5.5 (2.3) of 20 and a median of 5.5 (IQR, 3.0-7.0). At the posttest, the intervention group’s mean (SD) score was 9.6 (4.4) of 20 with a median of 9.0 (IQR, 6.8-13.0) The final test scores for the intervention group were a mean (SD) of 17.0 (2.2) of 20 and a median of 17.3 (IQR, 16.1-18.8). The control group had a pretest mean (SD) score of 5.5 (2.8) of 20 and a median of 5.0 (IQR, 3.0-8.0). The control group’s posttest scores were a mean (SD) of 7.6 (4.4) of 20 and a median of 7.0 (IQR, 4.0-10.0), and their final scores were a mean (SD) of 16.5 (3.7) of 20 with a median of 17.7 (IQR, 15.7-18.8).

Students’ mean (SD) scores in the preparticipation test were 5.5 [2.8] of 20 in the intervention group and 5.5 [2.3] of 20 in the control group (*P* = .47) (Figure). Students in the intervention group achieved notably higher mean (SD) scores in the postparticipation test compared with the control group (9.9 [2.1] of 20 vs 7.5 [2.4] of 20; *P* < .001). In contrast, no significant differences in mean (SD) scores were noted between the intervention and control groups in the final neuroanatomy examination, which took place 1 month later (17.2 [1.3] of 20 vs 16.5 [1.5] of 20; *P* = .09).

**Figure.**
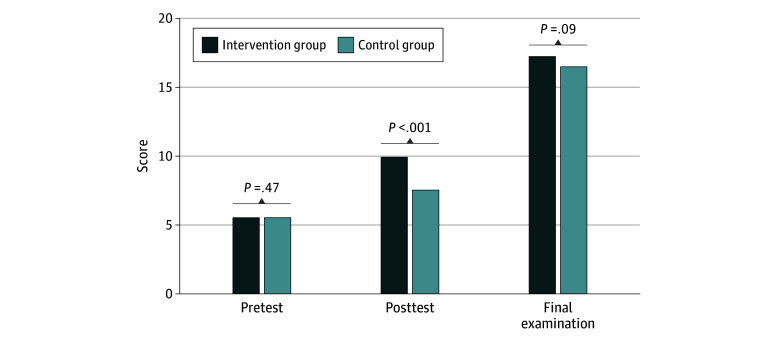
Scores in the Preparticipation and Postparticipation Tests and University Examinations

### Regression Analysis Findings

A multiple regression analysis was performed to assess the association of student group (intervention vs control), gender, previous anatomy grade, and grade point average with current neuroanatomy scores. The model indicated a weak but significant association between the variables and neuroanatomy scores (*R* = 0.342; *F* = 5.304; *P* < .001).

However, the model’s explanatory power was limited. The coefficient of determination (*R*^2^) was 0.117, with the adjusted *R*^2^ at 0.095, indicating that only 9.5% of the variance in neuroanatomy scores was explained by the variables in the model. Furthermore, the SE of the estimate was 20.71, highlighting a substantial margin of error in the estimation of individual scores.

An examination of the individual variables revealed that students in the control group had significantly higher scores, a mean of 10.6 (3.7) points above the intervention group (*P* = .001). Grade point average also had a significant positive association, where each 1-unit increase was associated with a 0.9-point increase in the neuroanatomy score (*P* = .002). Conversely, gender and previous anatomy grade did not have a statistically significant association with scores.

### Students’ Opinions and Satisfaction

Students’ opinions regarding the usefulness and benefits associated with the videos as a supplementary educational resource are summarized in Table 3. Prior to participating in the study, 133 of 167 students (79.6%) expected that viewers would gain scientific benefits from watching the videos, while 16 of 167 (9.6%) believed no such benefits would accrue. After participation, on the other hand, a smaller percentage of students (119 of 167 [71.3%]) continued to believe that viewers would gain scientific benefit, while more students did not expect any scientific benefit from the reels (26 of 167 [15.6%]).

**Table 3.** Students’ Opinions and Satisfaction Regarding Short Social Media Videos

Question	No. (%) of students in intervention group (N = 84)		
	Agree	Neutral	Disagree
It is better to watch the entire lecture?			
Preparticipation	17 (20.2)	21 (25.0)	46 (54.8)
Postparticipation	24 (28.6)	15 (17.9)	45 (53.6)
Do short videos help in attracting attention?			
Preparticipation	71 (84.5)	10 (11.9)	3 (3.6)
Postparticipation	74 (88.1)	7 (8.3)	3 (3.6)
Is watching reels useful for studying in general?			
Preparticipation	59 (70.2)	14 (16.7)	11 (13.1)
Postparticipation	60 (71.4)	16 (19.0)	8 (9.5)
Is watching reels a waste of time?			
Preparticipation	8 (9.5)	24 (28.6)	52 (61.9)
Postparticipation	9 (10.7)	15 (17.9)	60 (71.4)
Is watching reels essential for studying anatomy?			
Preparticipation	26 (31.0)	29 (34.5)	29 (34.5)
Postparticipation	29 (34.5)	28 (33.3)	27 (32.1)
Does using reels help study neuroanatomy?			
Preparticipation	43 (51.2)	30 (35.7)	11 (13.1)
Postparticipation	41 (48.8)	29 (34.5)	14 (16.7)

At the end of the study, 71 of 84 students (84.5%) in the intervention group indicated that watching the videos on neuroanatomy was helpful, while 11 of 84 viewers (13.1%) reported feeling uncomfortable when educational videos appeared during breaks or entertainment on the social media platform. Although most students (80 of 84 [95.2%]) expressed their desire to continue the use of educational videos in the anatomy laboratory, 23 of 84 students (27.4%) thought that videos were monotonous despite their richness in content.

## Discussion

Microlearning involves delivering informative content in small and manageable segments and is increasingly being embraced in educational environments, particularly by Millennial and Generation Z learners. One format of microlearning is short social media videos, which have the potential to enhance cognitive engagement and improve knowledge retention among students.^6^ The short video format has already been acknowledged as an important enhancement to traditional classroom instruction,^7,8,9,10^ as short videos can generate interest and expose learners to topics with quick and digestible content, matching the present models of attention.

The use of short social media videos in medical education can be especially useful in certain subjects, such as radiology,^11,12^ histology,^13^ and anatomy,^14,15,16,17^ where visual representation becomes crucial. In fact, a recent review of the use of these videos in anatomy education revealed that posts featuring illustrations consistently represented the most preferred format and that posts that incorporated humor experienced the most substantial increase in viewers among various post purposes.^16,17^

The present study suggests that the use of short social media videos is associated with significant short-term academic improvement. Students who engaged with the videos demonstrated significantly enhanced short-term recall compared with those relying solely on full lecture recordings, as evidenced by their markedly higher postparticipation test scores (9.9 of 20 vs 7.5 of 200; *P* < .001), despite both groups exhibiting identical baseline performance (5.5 of 20). This finding supports the assertion that condensed, visually engaging content can effectively bolster immediate comprehension in medical education settings. Although some students favored watching full lecture recordings, more students indicated that short videos may enhance engagement. It is likely that the brevity of social media videos supports microlearning,^6^ a strategy that has been shown to improve focus and engagement in medical education.^18^ The absence of significant differences in final examination scores between groups raises concerns about long-term retention and application of knowledge acquired through social media. Larsen et al^19^ have noted that spaced repetition and reinforcement are critical for long-term retention of knowledge, and spaced repetition and reinforcement were not integrated into the intervention in the present study.

The application of social media videos for educational objectives requires careful consideration of their drawbacks, such as the tendency toward passive learning and the need for dedicated personnel to manage their implementation.^16^ Institutions may be required to create specialized educational accounts to align student engagement with academic standards, as suggested by Shelton et al.^20^ This is consistent with broader educational research indicating that well-designed video instruction can be as effective as face-to-face methods, as seen in a study by Park et al^15^ where outcomes of video education on inhaler technique matched the outcomes of traditional teaching approaches. Furthermore, a concern arises regarding the proliferation of content, as many educational reels are prepared and circulated by peers, and other videos may have been made for advertising or profit purposes.^20,21^ Distinguishing reliable educational videos from noneducational posts on social media presents a considerable challenge.^7^ One example of noncontrolled social media content is that less than 32% of educational videos on acne treatment are created by certified dermatologists,^21^ and that information in many TikTok videos related to epicondylitis deviates from established medical guidelines.^22,23^ Although the use of reels in the present study was associated with positive student perceptions, most participants indicated that they preferred videos prepared by academic staff. Nevertheless, video production is fairly resource intensive (2-3 hours per 90-second reel), which raises scalability concerns and echoes challenges faced by faculty-led digital content creation.^24^ Academic institutions may also be required to establish specialized educational content creation centers to reduce faculty burden. Hybrid models that combine instructor-led reels with peer-generated content, paired with technical training, could ease resource strain. Success hinges on targeted design, quality control, and institutional support to preserve academic rigor while embracing innovation.

This study also provided insights into the challenges of integrating social media into pedagogy. Most students use social media as a means of entertainment and personal pursuits, but many are discomfited when academic content appears during nonstudy time. Disruption of leisure often contributes to cognitive overload or disengagement.^4^

### Limitations

This study has several limitations that should be considered. The primary limitation is the nonrandomized trial design, which introduces a significant risk of selection bias. The allocation of participants was based on voluntary self-selection and their preexisting access to Instagram. Consequently, students who were more motivated or already preferred learning via social media were more likely to join the intervention group. Although baseline academic performance was found to be comparable between the groups, we cannot fully distinguish whether the observed benefits are due to the intervention itself or to these preexisting motivational and learning-style preferences. As a result, the findings may have limited generalizability and are most applicable to learners already open to using social media for their studies.^25^

Furthermore, the measurement of the intervention’s exposure was a notable limitation. Engagement in the intervention group and adherence in the control group were based on self-reporting and could not be objectively verified. This creates a risk of misclassification and potential crossover between groups, meaning the observed associations should be interpreted with caution. The reliance on self-reported engagement metrics over the 2-month intervention period may not accurately reflect actual patterns of use. Future research should incorporate objective data, such as platform analytics, to address this issue.

## Conclusions

This nonrandomized clinical study suggests that short social media videos show promise for boosting students’ short-term engagement in anatomy education. Reels can act as a bridge between digital-native learners and traditional anatomy pedagogy, but the role of reels should complement—but not replace—formal curricula. The sustained impact of short social media videos demands ongoing evaluation, evidence-based modifications, and a focus on balancing creativity with educational outcomes.

## Notes

Supplement 1. **eFigure 1.** Examples of Questions Test **eFigure 2.** Screenshots Showcasing the Interface of the Instagram Account for the Laboratory of Anatomy at Damascus University (https://www.instagram.com/anatomy_damascus/), Along With Examples of Associated Reel Videos

Supplement 2.
Data Sharing Statement
