# Supplementary material for: Short Social Media Videos as a Supplementary Educational Resource in Neuroanatomy: A Nonrandomized Clinical Trial
Source: JAMA Netw Open. 2025 Sep 29;8(9):e2533971. doi: 10.1001/jamanetworkopen.2025.33971 (PMC12481223; doi:10.1001/jamanetworkopen.2025.33971)
Supplement: Supplement 1. — eFigure 1. Examples of Questions Test eFigure 2. Screenshots Showcasing the Interface of the Instagram Account for the Laboratory of Anatomy at Damascus University (https://www.instagram.com/anatomy_damascus/), Along With Examples of Associated Reel Videos [file jamanetwopen-e2533971-s001.pdf]

Supplementary Online Content

Alsaid B, Al-Bitar A, Mousa L, et al. Short social media videos as a supplementary educational resource in neuroanatomy: a nonrandomized clinical trial. *JAMA Netw Open.* 2025;8(9):e2533971. doi:10.1001/jamanetworkopen.2025.33971

eFigure 1. Examples of Questions Test

eFigure 2. Screenshots Showcasing the Interface of the Instagram Account for the Laboratory of Anatomy at Damascus University (https://www.instagram.com/anatomy_damascus/), Along With Examples of Associated Reel Videos

This supplementary material has been provided by the authors to give readers additional information about their work.

© 2025 Alsaid B et al. *JAMA Network Open.*

eFigure 1. Examples of Questions Test. A- multiple-choice questions (MCQs) with single answers, B- MCQs with multiple answers, C- pictures with MCQs to answer related information, and determine anatomical elements. D- arrow on pictures with MCQs to determine the anatomical element.

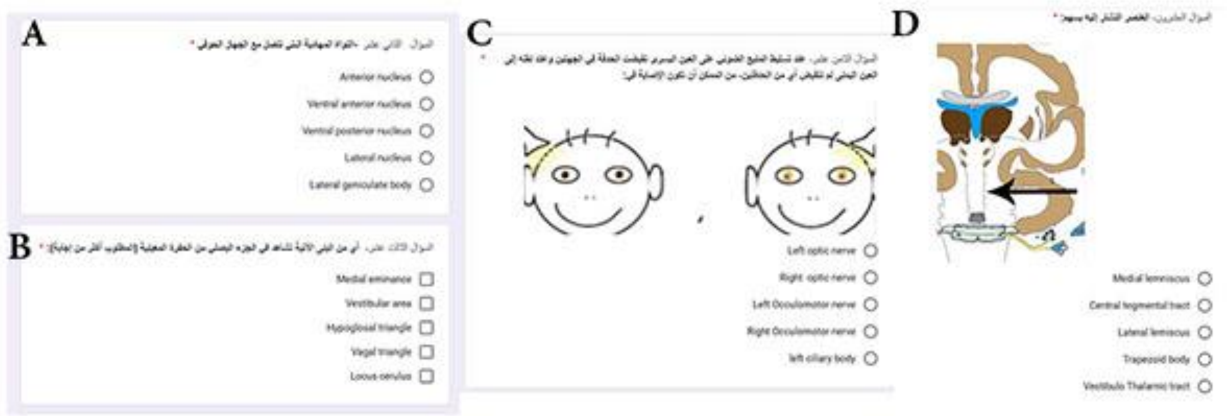

© 2025 Alsaid B et al. *JAMA Network Open.*

eFigure 2. Screenshots Showcasing the Interface of the Instagram Account for the Laboratory of Anatomy at Damascus University (https://www.instagram.com/anatomy_damascus/), Along With Examples of Associated Reel Videos

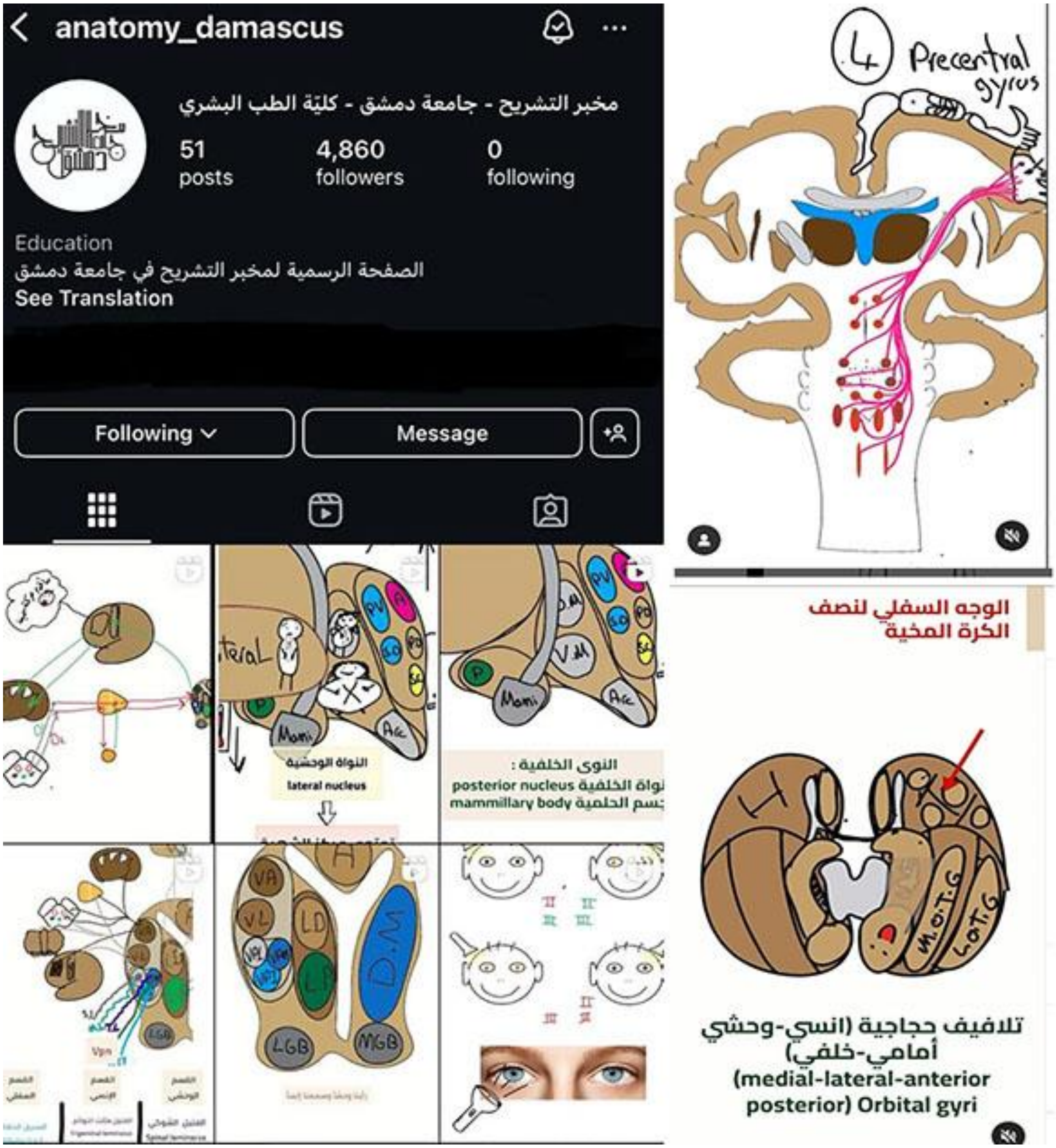

© 2025 Alsaid B et al. *JAMA Network Open.*
