# Supplementary material for: Short Social Media Videos as a Supplementary Educational Resource in Neuroanatomy: A Nonrandomized Clinical Trial
Source: JAMA Netw Open. 2025 Sep 29;8(9):e2533971. doi: 10.1001/jamanetworkopen.2025.33971 (PMC12481223; doi:10.1001/jamanetworkopen.2025.33971)
Supplement: Supplement 2. — Data Sharing Statement [file jamanetwopen-e2533971-s002.pdf]

Data Sharing Statement

Alsaid. Short Social Media Videos as a Supplementary Educational Resource in Neuroanatomy. *JAMA Netw Open*. Published September 29, 2025. doi:10.1001/jamanetworkopen.2025.33971

Data
Data available: No
